# Anti-Adenoviral Activity of 2-(3-Chlorotetrahydrofuran-2-yl)-4-Tosyl-5-(Perfluoropropyl)-1,2,3-Triazole

**DOI:** 10.3390/medicina54050081

**Published:** 2018-11-05

**Authors:** Liubov Biliavska, Yuliia Pankivska, Olga Povnitsa, Svitlana Zagorodnya, Ganna Gudz, Yuriy Shermolovich

**Affiliations:** 1 Zabolotny Institute of Microbiology and Virology, National Academy of Sciences of Ukraine, 154, Zabolotnogo str., 03143 Kyiv, Ukraine; pankivska.yulia@gmail.com (Y.P.); povnitsa@ukr.net (O.P.); svetazagorodnya@ukr.net (S.Z.); 2 Institute of Organic Chemistry, National Academy of Sciences of Ukraine, 5, Murmanska str., 02660 Kyiv, Ukraine; anutagudz@gmail.com (G.G.); sherm@ioch.kiev.ua (Y.S.)

**Keywords:** 1,2,3-triazole, human adenovirus, antiviral activity, cell cycle, protein kinase

## Abstract

*Background and objectives:* A considerable increase in the levels of adenoviral diseases among both adults and children necessitate the development of effective methods for its prevention and treatment. The synthesis of the new fluorinated 1,2,3-triazoles, and the study of the mechanisms of their action, are promising for the development of efficient antiviral drugs of our time. *Materials and Methods:* Antiviral activity and cell cytotoxic effect of 2-(3-chlorotetrahydrofuran-2-yl)-4-tosyl-5-(perfluoropropyl)-1,2,3-triazole (G29) were determined by MTT (3-(4,5-dimethylthiazol-2-yl)-2,5-diphenyl tetrazolium bromide) assay. The influence of the compound on the infectivity of human adenovirus type 5 (HAdV-5) was carried out via the cytomorphology method. The influence of the compound on the cell cycle under a condition of adenovirus infection was studied using flow cytometric analysis of propidium iodide-stained cells. *Results:* It was found that G29 suppressed HAdV-5 reproduction by 50% in concentrations of 37 μg/mL. Furthermore, the compound reduced the titer of virus obtained de novo, and inhibited HAdV-5 inclusion bodies formation by 84–90%. The use of fluorinated compounds under the conditions of adenovirus infection decreased the number of apoptotic cells by 11% and the number of cells in S phase by 21–42% compared to the profile of infected cells. *Conclusions:* The fluorinated compound G29 showed moderate activity against HAdV-5 based on several mechanisms. It led to the normalization of the life cycle of cells infected with adenovirus to the level of non-infected cells and caused the obstruction of HAdV-5 reproduction, inducing the formation of non-infectious virus progeny.

## 1. Introduction

Human adenoviruses (HAdVs) are characterized by the different degrees of pathogenicity and the course of the infectious process. An important socioeconomic aspect of adenoviral infection is associated with its ability to polyclinically manifest. From eye lesions, respiratory problems, gastrointestinal and urogenital tract infections, to a high percentage of mortality in immunosuppressive therapy after organ transplantation [1]. The treatment of adenoviral infections is controversial as currently no clinically approved antiviral drugs against HAdV exist. However, the anti-adenovirus activity of many compounds is investigated in vitro, where most of them are nucleoside and nucleotide analogs that are focused on blocking viral DNA polymerase [2]. The efficiency of cidofovir and ribavirin was clinically evaluated in the topical treatment of eye diseases and sуstemic treatment of life-threatening adenoviral infections. However, the results demonstrated some contradictions [3] as the use of the drugs in the treatment of disseminated adenoviral infection was often associated with low efficiency and resulted in the death of patients. Acceptable results were obtained in preclinical and clinical studies of brincidofovir, representing cidofovir derivatives that, compared to cidofovir, has higher biological activity and lower toxicity. However, the significant nephrotoxicity, suppression of the immune system, and the development of drug-resistant adenovirus strains associated with brincidofovir intake motivate further discovery of alternative anti-adenoviral drugs. Development of effective antiviral drugs is a difficult task because often genetically related types of adenoviruses can cause similar diseases [4].

Fluorinated analogs of biological molecules are useful tools for studying and modifying the functions of biological systems. They play a significant role in the treatment of cancer and viral infections as selective inhibitors of enzymes required for viral replication and as inducers of apoptosis, which causes their antitumor effect [5]. Much attention is paid to the synthesis of analogs of known biologically active substances modified by fluorine atoms because such fluorinated derivatives often exhibit higher action [6]. The introduction of fluorine atoms into biologically active molecules can affect, not only its pharmacokinetic properties, but also the distribution in tissue, the path, and the rate of metabolism of the fluorinated derivative and its pharmacodynamics and toxicology [7]. All these factors play a key role in the relevance of studies of the biological properties of fluorinated analogs of nucleotides and amino acids and, accordingly, the creation of drugs based on these compounds.

Therefore, the purpose of this work was to investigate the biological activity of the compound 2-(3-chlorotetrahydrofuran-2-yl)-4-tosyl-5-(perfluoropropyl)-1,2,3-triazole. Namely to study its effect on the viability and cell cycle of MDBK (the bovine kidney cells), and the reproduction of human adenovirus type 5.

## 2. Materials and Methods

### 2.1. Viruses and Cells

The MDBK cells and the HAdV-5, obtained from the tissue culture collection of the Institute of Virology of the Bulgarian Academy of Sciences (Sofia, Bulgaria) and collection of the Institute of Microbiology of the Budapest University of Medical Sciences (Budapest, Hungary), respectively, were used in this study and cultured according to standard methods [8,9].

### 2.2. Tested Substances

**Figure 1:**
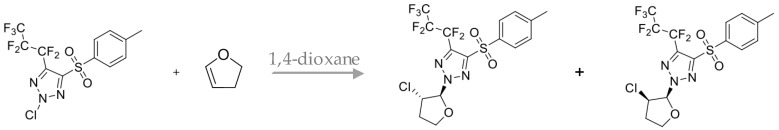
Scheme of synthesis and structure of the test compound.

Compound 2-(3-chlorotetrahydrofuran-2-yl)-4-tosyl-5-(perfluoropropyl)-1,2,3-triazole (G29) was synthesized at the Institute of organic chemistry of NAS of Ukraine [10]. The compound is heterocyclic in structure, composed of 3-chlorotetrahydrofuran residues in the glycoside portion of the molecule and heptafluoropropyl residues in the triazole ring (Figure 1). Ribavirin (Rib) was used as the reference drug.

Reagents and conditions: *N*-chlorotriazole reacts with 2,3-dihydro-2H-furan in 1,4-dioxane at room temperature, forming addition products to the double bond that are diastereoisomeric mixtures of 2-(cis-3-chlorotetrahydrofuran-2-yl)- and 2-(trans-3-chlorotetrahydrofuran-2-yl)-4-(heptafluoropropyl)- 5-tosyl-2H-1,2,3-triasoles. The mixtures of diastereoisomers were separated into individual isomers by column chromatography.

For prediction of the biological activity spectra of the compound were analyzed using the РАSS online web server [11].

### 2.3. Cellular Toxicity and Antiviral Assay

Cellular toxicity and antiviral activity of the compound evaluated by the inhibition of adenovirus cytopathic effect were tested in vitro according to the MTT-test [12]. Monolayers of МDBK cells in 96-multiwell plates were incubated with the compound at the concentrations of 8–1000 μg/mL for 48 h, then 20 μL of a 5 mg/mL solution of MTT (3-(4,5-dimethylthiazol-2-yl)-2,5-diphenyl tetrazolium bromide, Sigma, NY, USA) was added into the medium. Cells were incubated at 37 °C for 3 h, the medium was removed, and 150 μL of 96% ethanol was added. The plates were read using an automatic plate reader Multiskan FC (Thermo Scientific, Waltham, MA, USA) with a 538 nm test wavelength.

For the antiviral assay, 50 mL of virus suspension (a multiplicity of infection (MOI) of 3.5 PFU per cell) was added to the MDBK cells. After 1.5 h, any unabsorbed virus was aspirated and 200 µL of a serial two-fold diluted drug-containing medium was added to each well and incubated at 37 °C and 5% CO_2_. Further analysis was performed using standard MTT-method [13] after three days of incubation. The percent of protection was calculated by the following formula:

(OD exp.) − (OD virus control) (OD cell control) − (OD virus control) × 100%,

where OD exp. is the average optical density of treated infected cells, OD virus control is the average optical density of untreated infected cells, and OD cell control is the average optical density of treated uninfected cells. These indicate the absorbencies of the test sample, the virus control, and the cell control respectively.

### 2.4. Determination of the Infective Titer of Adenovirus Synthesized De Novo

MDBK cells were infected with HAdV-5 (at a MOI of 3.5). After adsorption, the compound at concentrations of 32–125 μg/mL was added. After 72 h, the influence of the compound on the infectivity of human adenovirus type 5 was tested via the cytomorphology method. The MDBK cells were grown to form a monolayer in test tubes with strips of cover coat (6 × 22 mm) and were then infected with 0.2 mL per test tube of ten-fold serial dilutions of the virus-containing suspension (treated or not treated with different concentrations of the compound). The virus adsorption was carried out for 1.5 h, then 0.8 mL of serum-free medium was added. Cells that were not infected with the virus were used as a control. After 48 h, cells were washed with Hanks solution (BioTestMed, Kiev, Ukraine) and fixed with 96% ethanol for 1 h. For microscopy, cells were washed with Hanks solution, stained with 0.01% solution of acridine orange (Sigma, Saint Louis, MO, USA) and examined through a fluorescent microscope (AmScope FM690TC, CA, USA) for the presence of virus-induced intranuclear inclusions (using the lens ×40). The number of inclusion-forming cells was determined and a decrease of virus titer synthesized de novo was calculated [14,15]:

*Virus titer: IFU/mL = (A* × *B*)/*C* where A is the total number of cells with inclusions in the test tube, B is the investigated dilution of the virus (opposite), and C is the volume of the inoculum.

% *Percentage inhibition of virus reproduction* = (1 − *T*/*C*) × 100%,

where *T* is the antilog of the compounds-treated viral titers and *C* is the antilog of the control (without compounds) viral titers.

### 2.5. Cell Cycle Research by Flow Cytometry

HAdV-5 infected cells (1 × 10^6^) treated and not treated with the compound were harvested by centrifugation at 300 *g* (2000 rpm) for 7 min, fixed in 96% ice-cold ethanol for 1 h, resuspended in 300 µL solution of phosphate-buffered saline (PBS) that contained RNAse (100 µg/mL) and propidium iodide (PI) (50 µg/mL), and incubated at 20 °C for 1 h [16,17]. The cell fluorescence intensity was measured by a flow cytometer (Beckman Coulter Epics LX, Minneapolis, MN, USA) with a laser wavelength of 488 nm. Cell cycle profiles were analyzed with the program Flowing Software, version 2.5 (Cell Imaging Core, Turku Centre for Biotechnology, Turku, Finland).

### 2.6. Statistical Analysis

Each study was conducted in at least three independent repetitions. Statistical data processing was performed according to standard approaches for calculating statistical errors (standard deviation) using Microsoft Excel 2010. The concentrations of the compound that inhibit cells viability by 50% compared to control cells (CC_50_) and inhibit virus reproduction by 50% compared to virus control (IC_50_) were determined. The results were expressed as mean ± S.D. for three independent experiments. Student’s unpaired *t*-test was used to evaluate the difference between the test sample and control. A *p* value of <0.05 was considered statistically significant.

## 3. Results

**Figure 2:**
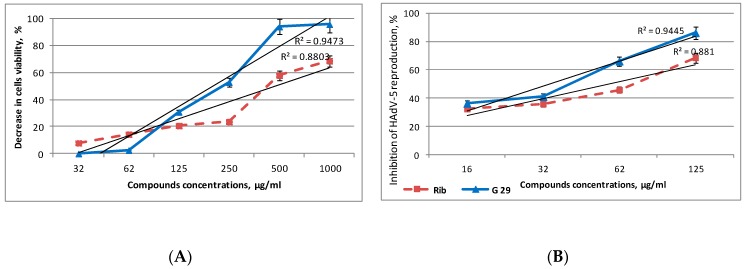
The effects of the compound G29 and Rib on the viability of MDBK cells (**A**) and HAdV-5 reproduction (**B**). Antiviral activity and cell cytotoxic effect were determined by MTT assay. Each point represents the mean value ± S.D. standard deviation (error bars) of the three independent experiments.

The analysis of compound G29 concentrations that are nontoxic for sensitive to human adenovirus 5 MDBK cells (Figure 2A) showed that the compound possesses significant cytotoxicity at concentrations of 500 and 1000 μg/mL, as cell viability decreased by more than 90%. Using the linear regression model in Microsoft Excel (predictor function) and dose-dependent values of the compound cytotoxicity, it was estimated that the CC_50_ index of compound G29 equaled 222 μg/mL. The compound concentrations that did not lead to visible and significant changes in cell morphology or density were used for the estimation of anti-adenovirus activity. Using MTT assay it was revealed that G29 at concentrations of 32–125 μg/mL inhibited adenovirus reproduction in cells by 38–84% and suppressed the development of adenovirus cytopathic effect (CPE) by 50% at the concentration of 37 μg/mL.

**Figure 3:**
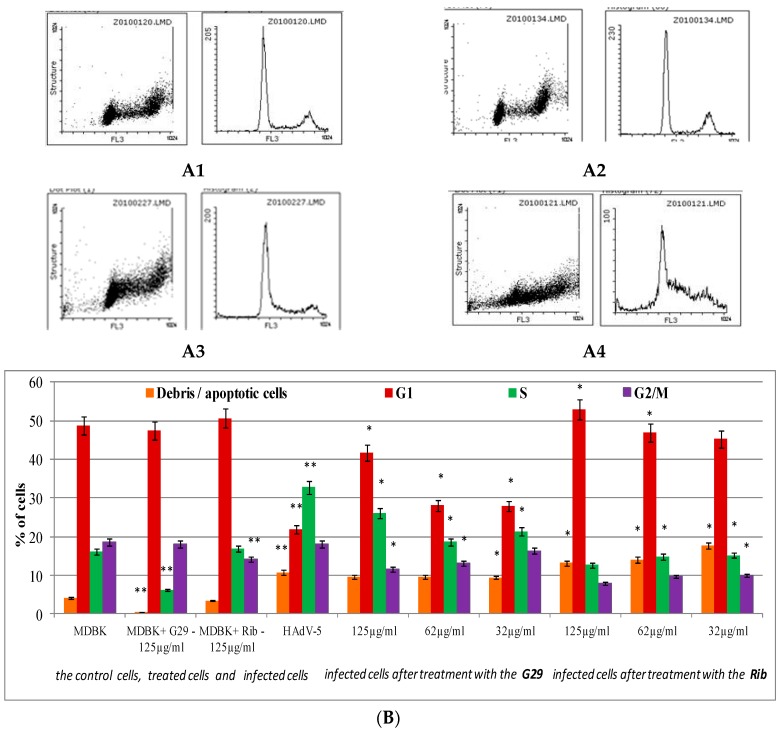
The influence of the compound G29 on the cell cycle of the MDBK cells in the presence and absence of adenovirus infections. (**A1**)—Cell cycle profile of the control cells; (**A2**,**A3**)—cell cycle profiles of treated cells (the compounds were used in the highest concentration used for the analysis of antiviral activity); (**A4**)—cell cycle profiles of cells infected with adenovirus. (**B**) Cell cycle features of infected cells after treatment with the compounds were measured by flow cytometry after staining with propidium iodide. Cell cycle profiles were analyzed with the Flowing Software, version 2.5 (Cell Imaging Core, Turku Centre for Biotechnology, Turku, Finland). Results corresponding to the percentage of cells in G1, S, and G2/M phases of three independent experiments are presented as mean ± S.D. * Significant difference between test sample and control of infected cells (*p* < 0.05). ** Significant difference between a test sample and control cells (*p* < 0.05).

Suppression of cell growth and proliferation frequently represents cell response to compound cytotoxicity and virus infection. Therefore, the influence of G29 on the cell cycle under normal conditions and the conditions of adenovirus infection was analyzed. For the test’s purpose, cells were fixed and stained with fluorochrome propidium iodide (PI) that intercalates into DNA. As the intensity of the PI signal is directly associated with DNA content, the number of cells in a certain cell cycle phase and cells containing fragmented DNA (apoptotic cells), as well as cell structure, were estimated. The typical cell cycle is observed in Figure 3(A1). In these conditions, a majority of cells are in G0/G1 phase (the highest pick), another pick represents cells in G2/M phase, and the area between two picks shows cells in S-phase. The effect of the G29 compound on MDBK cells population is demonstrated on the following histogram (Figure 3(A2)). As can be seen from the distribution of cells according to the structure and cell cycle phase, the histograms are similar but not identical. It was revealed that after 48 h of growth, 49% of MDBK cells remained in G1 phase, 16% were in S phase, and 18% were in the G2/M phase of cell cycle (Figure 3B). Under the conditions of the compound treatment, distribution of cells in G1 and G2/M phases was similar to control cells. However, there was a decrease in the number of cells in S phase by 60%.

Significant changes in the life cycle of cells infected with adenovirus were observed. As a consequence, adenovirus infection caused an increase in the number of apoptotic cells by 11% and increased cells in S phase by 33%, which is associated with enhanced G1 to S phase transition (Figure 3A,B). The decrease in the number of cells in G1 phase by 55% reveals that the transition through the mitotic phase of a cell cycle is inhibited. During the S to G2/M transition, synthesis of viral DNA, late viral proteins, and virions occur. Later, these cells die and detach from monolayer remaining in the medium.

It was demonstrated that the use of fluorinated compound under the conditions of adenovirus infection decreases the number of apoptotic cells by 11% compared to virus control (Figure 3B). The effect of G29 on the cell cycle during the infection was shown to be dose-dependent, as a decrease in the compound concentration was associated with the transition of cells from G1 to S1 and G2/M phases and enhanced replication of viral DNA. The use of the compound increased the number of cells in G1 phase by 15–43% and decreased the number of cells in S phase by 21–42% compared to the profile of infected cells, suggesting the normalization of the cell cycle of the infected cell to the level observed in non-infected cells.

**Figure 4:**
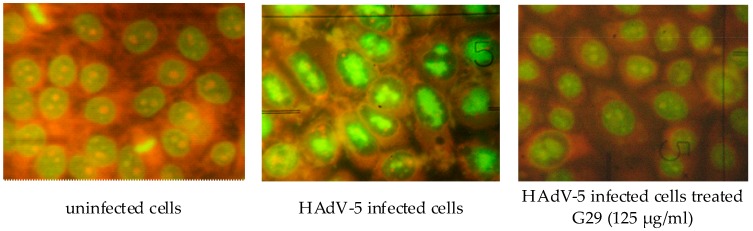
Cytomorphological features of virus infection in MDBK cells (acridine orange staining, magnification ×280). Adenoviral inclusions in the cells have the form of granular or center-nucleus bright green, surrounded by a dark, homogeneous zone.

As the decrease in virus CPE and normalization of the cell cycle observed in cells treated with G29 does not indicate the quality of virus production (for instance, the viral infectivity), the estimation of de novo produced adenovirus titer in the presence of different compound concentrations was performed. The influence of the compound on the infectivity of human adenovirus type 5 was carried out with cytomorphology method. It was used to identify infected cells containing specific intranuclear inclusion bodies induced by the virus, which can be detected with fluorescent microscopy after staining of fixed cells with acridine orange (Figure 4).

The number of inclusion-forming cells was determined, and a decrease of virus titer was calculated. Compound G29 showed significant antiviral activity as all analyzed concentrations decreased the infectious titer of adenovirus by 84–90% (Table 1).

## 4. Discussion

It is known that fluorinated analogs of nucleotides possess various biological activities comprising antiviral, antibacterial, antitumor, and antioxidant properties [18]. Consequently, the expansion of the antiviral spectrum of the given class of compounds, and examination of their activity against HAdV-5, is a crucial subject of the antiviral researches.

Previously, the antiviral properties of the several compounds derived from 4-tosyl-5-polyfluoroalkyl-1,2,3-triazoles (G16, G18, G19, and G29) were studied. These are heterocyclic compounds composed of 3-chlorotetrahydrofuran (G29), 2-deoxy-2-chloro-β-D-arabinofuranosyl (G16) or β-D-ribofuranosyl (G18 and G19) residues in the glycoside portion of the molecule, and difluoromethyl (G19), trifluoromethyl (G18), perfluoropropyl (G16), and heptafluoropropyl (G29) residues in the triazole ring. However, we found that compounds G16, G18, and G19 were not active against adenovirus (data not shown). Based on these results, G29 was selected for further research.

The current work explores the biological activity of a compound 2-(3-chlorotetrahydrofuran-2-yl)-4-tosyl-5-(perfluoropropyl)-1,2,3-triazole that represents the analog of Ribavirin. The results of the MTT analysis demonstrate the apparent anti-HAdV-5 action of the compound, as it inhibited the development of adenovirus CPE by 42–84% at the concentrations of 32–125 μg/mL.

Virus infection frequently results in the disturbance of key cellular processes within the host cell. The subversion of cell cycle pathways is a well-established mechanism by which viruses create the most suitable environment for their replication [19]. Notably, the induction of S-phase is either mandatory or at least advantageous for lytic replication of a number of viruses. The adenoviral infection has been reported to have effects on the cell cycle. It is well-known that adenoviral E1 gene products interact with pRb (retinoblastoma protein), causing the release of E2F transcription factor, which potentiates transition from G1 to S phase, in which productivity is greatest. HAdV infection of a range of epithelial cell lines, including a primary cell line, causes G2 phase synchronization and cell cycle arrest [20,21]. This synchronization in the G2 phase may be a significant factor contributing to the cell-size increase [21].

The characteristic changes in DNA synthesis and content induced by HAdV-5 infection allow the use of flow cytometry to detect not only viral infection, but also the potential antiviral activities [21]. The influence of the compound G29 on the cell cycle under a condition of adenovirus infection was studied using flow cytometric analysis of propidium iodide-stained cells. The normalization of the number of cells in all phases of the cell cycle compared with the profile of infected cells, and the increasing number of cells in G1 phase to 43% compared with the control values of viral infections, were determined after using G29.

In addition, using the cytomorphology method based on the detection of infected cells containing specific intranuclear inclusion bodies induced by the HAdV-5, the influence of the compound on the formation of the infectious progeny of adenovirus was studied. It was found that the compound G29 at the concentration of 32–125 μg/mL inhibited HAdV-5 inclusion-bodies formation by 90%.

Fluorinated analogs of nucleosides/nucleotides and 1,2,3-triazoles derivatives are widely used in clinics for the treatment of socially significant diseases caused by dangerous viruses including human immunodeficiency virus, herpes simplex viruses 1 and 2, cytomegalovirus, hepatitis B and C viruses, etc. [22,23]. These compounds represent chemically modified mimetics of natural nucleosides that, as the result of biotransformations in cells, are inserted into DNA or RNA and suppress virus replication or cell proliferation. Moreover, these compounds may interact with and inhibit essential enzymes that regulate nucleic acid metabolism including virus and cell nucleotide polymerases, kinases, ribonucleotide reductases, DNA-methyltransferases, purine and pyrimidine nucleoside phosphorylases, etc. [7].

According to the performed bioinformatic analysis of the biological activity of the compound using the PASS system, G29 might serve as an inhibitor of protein kinase CK2 (Pa/Pi values equaled 0.405/0.003). It is known that the normal functioning of a cell requires coordinated regulation of growth, proliferation, and differentiation controlled at the molecular level. Protein kinase CK2 is a crucial link in numerous cellular signaling pathways including transcription, translation, cell cycle control, proliferation, cell survival, and apoptosis [24]. Ribosomal RNA transcription is one of the critical factors required for cell survival. The process is enhanced by CK2, which interacts with complex Pol1/Rrn3 (Rrn3-RNA polymerase I-specific transcription initiation factor), recruits it to promoter regions of rRNA genes, and maintains a high activity of RNA polymerase 1 providing rRNA synthesis [24]. Moreover, CK2 is involved in the regulation of apoptosis related to phosphorylation of p53 oncosuppressor, which binds to regulatory regions of DNA activating promoters. Gene encoding protein p21 WAF1 is one of the targets of p53-dependent processes and its activation leads to the inhibition of cell growth due to the G1/S transition block [25].

Much attention has been drawn to the search for the drugs among nucleosides derivatives (e.g., β-D-ribofuranozilbenzimidazol) to fight against human cytomegalovirus (HCMV) and HIV-1 over the last decade. It was shown that the mechanism of their action was based on the inhibition of CK2, which indirectly enhanced HIV-1 and HCMV gene expression at the transcriptional level. As a result, CK2 inhibitors are used as suppressors of viral gene transcription [26,27]. The number of known viral proteins that are the substrates of the CK2 kinase is rapidly increasing. Therefore, CK2 is a potential target for the drugs that can be used in medical therapy.

## 5. Conclusions

Taken together, the compound G29 possessed distinct anti-HAdV-5 activity that was based on several mechanisms. As a result, the compound led to the normalization of the life cycle of cells infected with adenovirus to the level of non-infected cells and caused the obstruction of HAdV-5 reproduction, inducing the formation of non-infectious virus progeny. On the basis of the obtained results, it can be suggested that the compound may inhibit protein kinase CK2, probably resulting in the alteration of the life cycle of infected cells and the suppression of virus transcription, translation, and virion assembly.

## Figures and Tables

**Table 1:** Effect of G29 on the formation of infectious progeny of adenovirus.

Concentration of the Compound, μg/mL		Titer of HAdV-5, IFU/mL	Inhibition of HAdV-5 Reproduction, %
**G29**	125	1.2 × 10^5^	90.34
	62	1.5 × 10^5^	88.26
	32	2 × 10^5^	84.43
**Rib**	125	2 × 10^3^	99.84
	62	2.3 × 10^4^	98.23
	32	4.8 × 10^4^	96.30
Virus control		1.3 × 10^6^	-

*Note:* G29 (32–125 µg/mL) was added to monolayers of MDBK cells after infection with HAdV-5 (at a MOI of 3.5). At 72 h after infection, all cultures were harvested and virus titer was determined by cytomorphology method. The MDBK cells were then infected with ten-fold serial dilutions of the virus-containing suspension (treated or not treated with different concentrations of the compound). After 48 h, cells were fixed with 96° ethanol, stained with 0.01% solution of acridine orange, and examined with a fluorescent microscope for the presence of virus-induced intranuclear inclusions.
